# Unlocking an acute psychiatric ward: open doors, absent patients?

**DOI:** 10.1192/bjb.2018.35

**Published:** 2018-06

**Authors:** Damian Smith, MacDara McCauley

**Affiliations:** Louth Meath Mental Health Services and Royal College of Surgeons Ireland; email: damiansmith@rcsi.com

In their recent paper, Beaglehole and colleagues^1^ reported on the effects of unlocking an acute psychiatric ward. Despite a 58% increase in unauthorised absences and an 8% increase in violent incidents, they concluded that a less restrictive environment had some positive effects, most notably a reduction in the total hours of seclusion per month.

Our service has recently undertaken a similar transition from a locked acute ward opened (and locked) in the 1930s, to an unlocked newly built unit opened in 2016. When comparing the 6 months before and after this transition, we too found that the rate of unauthorised absences increased by 100% from a mean of 4 to 8 per month. Unlike Beaglehole, however, we observed a decrease in rates of violent incidents by 27.4% (from a mean of 31.7 to 23 per month), and an increase in the total hours of seclusion per month by 213.4% (from a mean of 28.21 to 88.42 hours per month). Of note, admission rates increased from a mean of 20 to 23 per month during the same time period.

Although a reduction in the rate of violent incidents (and, in the case of Beaglehole, reduced levels of seclusion) strengthens the case for provision of care in unlocked settings, should we be concerned about the increased rate of unauthorised absences found in both studies?

The largest available study on this topic^2^ would suggest not. In their 15-year observational study involving 145,738 German in-patients, Huber *et al* concluded that locked doors do not prevent suicides, or indeed unauthorised absences.

Although a rare event, suicide is undoubtedly one the most feared outcomes when any patient absconds. Preventing harm to self or others is often the main rationale for in-patient admission. It is also a ubiquitous criterion for involuntary admission. Consequently, preventing harm is one of the main motives for locking psychiatric units.

In our study, 86% of unauthorised absences over the 1-year study period were by involuntarily admitted patients. In opening our doors, are we doing these individuals a disservice by giving them the opportunity to leave hospital at a time when they are most unwell?

Previous studies have reported on the negative consequences of absconding for patients (interrupted treatment, suicide), staff (anxiety), family members (loss of trust in the service), and emergency services (expended resources).^3^ It could be argued that a reduction in the number of violent incidents (and, in Beaglehole's case, seclusion) is worth the risk of these adverse outcomes. In our view, however, a modern purpose-built environment coupled with increased staffing levels better explains these findings. Increased numbers of nursing staff result in improved relational security, an important element of therapeutic security provided by higher staff-to-patient ratios.^4^

Our study and that of Beaglehole and colleagues indicate that unlocking acute psychiatric wards leads to an upsurge in unauthorised absences. The majority of patients who absconded were admitted involuntarily. We suggest that acute mental health services give careful consideration to all the risks associated with unauthorised absences before opening their doors. This is of particular relevance given that rates of involuntary admission are on the rise.^5^
